# Rapid evolution of quantitative traits: theoretical perspectives

**DOI:** 10.1111/eva.12127

**Published:** 2013-12-06

**Authors:** Michael Kopp, Sebastian Matuszewski

**Affiliations:** 1 LATP UMR-CNRS 7353, Evolutionary Biology and Modeling Group, Aix Marseille UniversityMarseille, France; 2 Mathematics and BioSciences Group, Faculty of Mathematics, University of ViennaVienna, Austria

**Keywords:** Adaptation, climate change, habitat degradation, natural selection and contemporary evolution, phenotypic plasticity, population dynamics, population genetics, quantitative genetics

## Abstract

An increasing number of studies demonstrate phenotypic and genetic changes in natural populations that are subject to climate change, and there is hope that some of these changes will contribute to avoiding species extinctions (‘evolutionary rescue’). Here, we review theoretical models of rapid evolution in quantitative traits that can shed light on the potential for adaptation to a changing climate. Our focus is on quantitative-genetic models with selection for a moving phenotypic optimum. We point out that there is no one-to-one relationship between the rate of adaptation and population survival, because the former depends on relative fitness and the latter on absolute fitness. Nevertheless, previous estimates that sustainable rates of genetically based change usually do not exceed 0.1 *haldanes* (i.e., phenotypic standard deviations per generation) are probably correct. Survival can be greatly facilitated by phenotypic plasticity, and heritable variation in plasticity can further speed up genetic evolution. Multivariate selection and genetic correlations are frequently assumed to constrain adaptation, but this is not necessarily the case and depends on the geometric relationship between the fitness landscape and the structure of genetic variation. Similar conclusions hold for adaptation to shifting spatial gradients. Recent models of adaptation in multispecies communities indicate that the potential for rapid evolution is strongly influenced by interspecific competition.

## Introduction

Over the past two decades, it has become clear that evolutionary change can be fast enough to be observed in present-day populations (Hendry and Kinnison 1999; Kinnison and Hendry 2001; Hendry et al. 2008; Gingerich 2009) and that it can directly affect the dynamics of populations and communities (Hairston et al. 2005; Saccheri and Hanski 2006; Kinnison and Hairston 2007; Pelletier et al. 2009). Much recent interest has focused on the possibility that so-called rapid or contemporary evolution leads to ‘evolutionary rescue’, whereby threatened populations avoid extinction by adapting to an altered environment (Barrett and Hendry 2012; Gonzalez et al. 2013). This issue is particularly pressing in the context of global climate change, which subjects large numbers of populations to shifts in temperature, aridity, seasonal patterns, etc. While phenotypic responses to climate change have been documented (Bradshaw and Holzapfel 2006; Parmesan 2006; Hoffmann and Sgro 2011 and this issue), the potential for evolutionary rescue is still unclear (Bell 2013). At the same time, it is often difficult to distinguish changes based on genetic evolution from those due to phenotypic plasticity (Merilä 2012; Merilä and Hendry 2014).

At the basis of many questions in the context of adaptation to environmental change are rates of phenotypic evolution (Hendry and Kinnison 1999; Kinnison and Hendry 2001; Gingerich 2009). These rates are often measured in *haldanes*. One *haldane* is equivalent to a change in one phenotypic standard deviation per generation (for other measures, see discussion in Hendry and Kinnison 1999, and for alternative standardizations and issues of scale, Hereford et al. 2004; Hansen and Houle 2008). Several recent meta-analyses of contemporary evolution yield the following picture: evolutionary rates above 0.1 *haldanes* are not uncommon (Hendry and Kinnison 1999; Gingerich 2009), even though the majority of rates are lower (Kinnison and Hendry 2001). Rates are higher in populations that are strongly influenced by human activities (Hendry et al. 2008; Darimont et al. 2009). Rates measured over few generations are higher than those measured over many generations (Gingerich 1983; Kinnison and Hendry 2001; Hendry et al. 2008; Gingerich 2009; Westley 2011). Studies that controlled for environmental effects (e.g., using common garden experiments) find lower rates than those that do not (Hendry et al. 2008), suggesting a role for phenotypic plasticity (Pigliucci and Murren 2003; Hendry et al. 2008; Westley 2011). Over paleontological timescales, the best-fitting model of phenotypic evolution is one of stasis interrupted by bursts of change (Estes and Arnold 2007; Uyeda et al. 2011).

The aim of this study is to review quantitative-genetic models that shed light on the potential for rapid adaptation. Our focus will be on the evolution of quantitative traits, that is, traits with continuous variation that are determined by a large number of loci with appreciable standing genetic variation. While we will frequently mention the link between adaptation and population survival, we do not aim for a comprehensive review of evolutionary rescue theory (see Gonzalez et al. 2013 and 14 other articles in a recent theme issue of the *Philosophical Transactions of the Royal Society B*, vol. 368:1610). In particular, we will not treat evolutionary rescue via the fixation of single large mutations (Gomulkiewicz and Holt 1995; Holt and Gomulkiewicz 1997; Orr and Unckless 2008; Uecker and Hermisson 2011; Kirkpatrick and Peischl 2013; Martin et al. 2013).

The structure of the article is as follows. We first give a detailed description of the basic models of adaptation of single and multiple quantitative traits under various scenarios of environmental change, including a discussion of ‘maximal sustainable rates of evolution’ (Bürger and Lynch 1995). Subsequently, we discuss four avenues into which the basic models have been extended by recent work (i) the role of phenotypic plasticity and its interactions with genetic evolution, (ii) determinants of adaptive potential and evolvability, (iii) adaptation to shifting spatial gradients and (iv) evolution and adaptation in a community context.

## Basic models

### Modeling approaches

#### Environmental change

**Figure 1:**
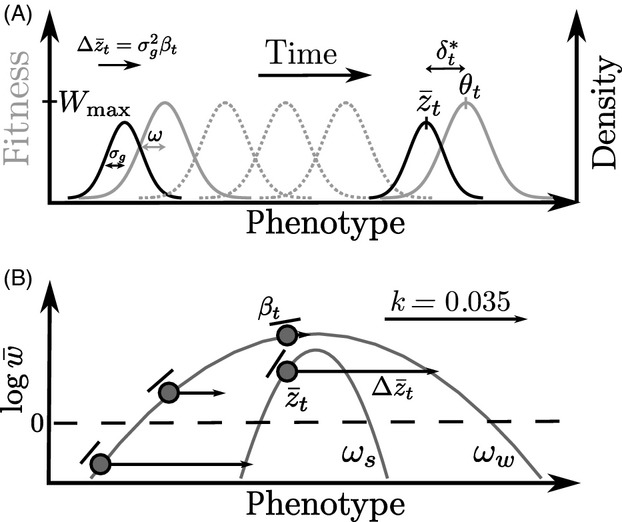
Illustration of trait evolution in the one-dimensional moving-optimum model. (A) Solid and dotted gray curves represent the fitness landscape at different points in time (eq. 2), whose width is determined by *ω*. *θ*_*t*_ is the optimal phenotype, which moves at constant speed (*θ*_*t*_ = *kt*). The black curves represent the distribution of breeding values in the population (mean 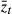, variance 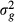). The mean phenotype evolves according to eqn (1). At the dynamic equilibrium, it follows the optimum with a constant lag 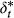. (B) illustrates the relation between rate of evolution and extinction risk. The gray curves show the log mean fitness as a function of the mean phenotype 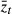 for two different fitness functions with widths *ω*_*s*_ and *ω*_*w*_, respectively. The rate of evolution, given by the horizontal arrows is determined by the fitness gradient *β*_*t*_, indicated by the black lines. The vertical position of the population gives its mean (log) fitness. In the Figure, the optimum is assumed to move at rate *k* = 0.035, and the population placed at the narrow fitness curve follows at this pace while maintaining a positive growth rate (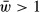). With the wide fitness function, however, the same rate of evolution requires a larger distance from the optimum, such that the growth rate is negative and the population goes extinct.

Most theoretical approaches to adaptation in a changing environment are based on models of stabilizing selection with a moving optimum. That is, at any given time, selection favors a specific trait value (or combination of trait values), but this favored phenotype changes over time. The most important scenarios are the following:

- A single, sudden change in the optimum: this is a classic scenario studied in population genetics and also in recent models about the genetic basis of adaptation (Orr 2005) and evolutionary rescue (Orr and Unckless 2008). It is well suited to study adaptation in invasive species, as well as in species suffering a sudden degradation of their environment.
- Gradual (typically linear) movement of the optimum: this scenario seems best suited to investigate the effects of continued climate change (Fig. 1).
- Random fluctuations of the optimum, either around a constant value or around a linear trend: these fluctuations may or may not show autocorrelation. Such models are useful to study the effects of environmental stochasticity that overlay all climate-driven trends.

#### Genetic adaptation

The majority of models reviewed here are based on quantitative genetics theory. Evolving traits are assumed to have a polygenic basis and follow a normal distribution with phenotypic variance 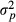. In the simplest case (additive genetics, no phenotypic plasticity), 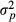 can be decomposed into 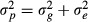, where 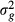 is the additive genetic variance, 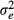 is the environmental variance (variation due to developmental instability and micro-environmental fluctuations), and 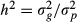 is the (narrow-sense) heritability. If phenotypes are measured in units of the environmental variance, 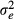 can be set to 1 (e.g., Bürger and Lynch 1995). The key theoretical tool for studying phenotypic evolution is the Lande equation (Lande 1976a), whose univariate version reads


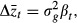
(1)

where 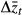 is the change in mean phenotype after one generation of selection, and 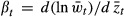 is the selection gradient at time *t*, that is, the derivative of log mean fitness 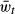 with respect to the mean phenotype. Note that eqn (1) is analogous to the univariate breeder's equation 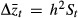, where 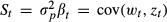 is the selection differential. A rate of change in *haldanes* can be obtained by standardizing with *σ*_*p*_, yielding


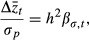
(2)

where *β*_*σ*,*t*_ = cov(*w*_*t*_, *z*_*t*_)/*σ*_*p*_ is the variance-standardized selection gradient (Lande and Arnold 1983; Hereford et al. 2004).

**Figure 2:**
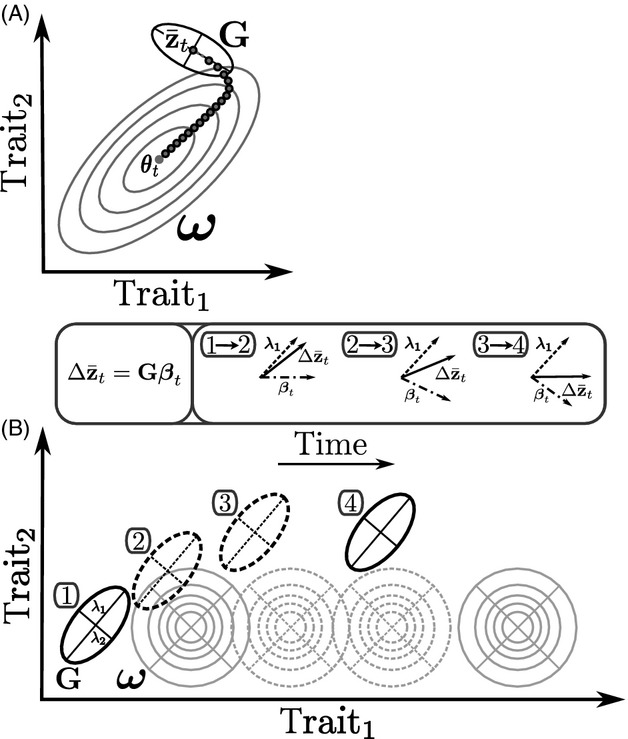
Illustration of adaptation involving two genetically correlated traits. (A) Adaptation after a sudden environmental change; the new optimum ***θ***_*t*_ is constant. Gray lines illustrate the fitness surface, defined by the matrix ***ω***. The distribution of breeding values defined by the **G**-matrix is illustrated by the black ellipse, whose center is the mean phenotype 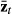 and whose axis is the eigenvectors of **G**. The initial response to selection is biased toward the leading eigenvector, that is, the genetic line of least resistance (Schluter 1996). (B) Adaptation to a moving optimum. Gray circles show the fitness landscape at four different points in time. Black ellipses show the corresponding positions of the population (represented by the **G**-matrix). The insets at the top show the leading eigenvector of **G**, *λ*_1_, the selection gradient ***β***_*t*_ and the response to selection 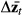 at time points 1, 2, and 3, respectively. Because the initial response is biased toward the leading eigenvector, the population ‘rises’ above the line of the moving optimum (i.e., the flying-kite effect; Jones et al. 2004). This rise comes to a halt as the tendency to follow the line of least resistance is balanced by the selection gradient, resulting in horizontal movement of the population.

For multiple traits, the structure of phenotypic variation is summarized by the matrix **P**, whose diagonal entries contain the phenotypic variances of the individual traits, and whose off-diagonal entries contain the phenotypic covariances. In the standard model, **P** = **G**+**E**, where **G** is the (additive) genetic covariance matrix and **E** the matrix of environmental variances and covariances. The multivariate version of Lande's equation is


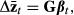
(3)

where, for *n* traits, 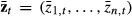
′ is the vector of mean trait values (with ′ denoting transposition) and 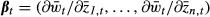
′ is the multivariate selection gradient, which points in the direction of steepest ascent on the fitness landscape. The response to selection is also influenced by the structure of genetic variation specified in the **G**-matrix. In particular, genetic correlations can cause the response to selection to show a bias toward trait combinations with high genetic variation (see Fig. 2 below; for an introduction to the geometric aspects of multivariate selection, see Walsh and Blows 2009).

The structure of multivariate genetic variation is often analyzed in terms of the eigenvectors of the **G**-matrix (as in a principal component analysis). The eigenvectors (principal components) can be viewed as composite traits (linear combinations of the original traits) that are genetically uncorrelated (i.e., their covariances are zero) and whose genetic variances are given by the corresponding eigenvalues. Graphically, if the distribution of breeding values (i.e., the average contribution of an individual to the phenotype of its offspring) is multivariate Gaussian, isoclines of this distribution can be represented by ellipses (or higher-dimensional ellipsoids), with axes given by the eigenvectors and their lengths proportional to the roots of the eigenvalues (Fig. 2). The major axis of such an ellipse (i.e., the leading eigenvector of the **G**-matrix) represents the trait combination with a maximum of genetic variation. Is has been called **g**_max_ or the *genetic line of least resistance* (Schluter 1996). Eigenvectors with small (or zero) eigenvalues represent trait combinations with little (or no) genetic variation, into which evolution is severely constrained (Hansen and Houle 2008; Gomulkiewicz and Houle 2009; Kirkpatrick 2009; Walsh and Blows 2009; Chevin 2013). More generally, it is also possible to calculate the amount of variation along any direction of the phenotypic space (Hansen and Houle 2008; Gomulkiewicz and Houle 2009). For the pros and cons of multivariate analysis in quantitative genetics, see Houle et al. (2002), Mezey and Houle (2003), Pigliucci and Kaplan (2006), Blows (2007), Walsh and Blows (2009), Berner (2012) and the commentaries to Blows (2007) in volume 20:1 of the *Journal of Evolutionary Biology*.

#### Phenotypic plasticity

Phenotypic plasticity in quantitative traits is usually characterized by *reaction norms*, which give the phenotype as a function of an environmental variable. When different genotypes have different reaction norms, plasticity is itself evolvable. While the evolution of plasticity can be modeled in different ways (Via and Lande 1985; De Jong 1995, see also Box 1 in Chevin et al. 2013), most of the models reviewed here focus on linear reaction norms and treat their slope and elevation as quantitative traits (e.g., Lande 2009). The majority of models have studied plasticity in single traits only (but see Gavrilets and Scheiner 1993; Draghi and Whitlock 2012), even though the **G**-matrix is known to be sensitive to environmental conditions (e.g., Tonsor and Scheiner 2007; Husby et al. 2011).

#### Population dynamics

Models of evolutionary rescue assume that the intrinsic population growth rate depends on the degree of adaptation, that is, on mean absolute fitness. Regardless of potential density dependence, a population will decline if the average number of offspring per individual drops below 1. That is, eventually, population size *N* is likely to follow


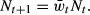
(4)

As shown in Appendix 1, the mean fitness 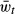 is generally reduced by two kinds of genetic load (Lande and Shannon 1996; Chevin 2013): a *standing load* due to phenotypic variation and a *lag load* (Maynard and Smith 1976) due to deviations of the mean phenotype from the optimum (also called selection load). In many models, survival or extinction of the population depends primarily on the lag load. A crucial point is that population dynamics depend on the mean fitness (eqn 4), whereas evolutionary change depends on the fitness gradient (eqns 1 or 3). Another way of saying this is that population dynamics depend on absolute fitness and evolution on relative fitness (Bell 2013). The relationship between these two quantities is determined by the fitness function: a given fitness gradient can be associated with a higher mean fitness under strong selection than under weak selection (Fig. 1B). This point will be essential in our discussion of sustainable evolutionary rates (see below).

Some predictions from the various models reviewed in this study are summarized in Table 1.

**Table 1:** A summary of theoretical predictions for models of adaptation to environmental change

Mode of Env. Change	Phenotypic Evolution	Survival/Extinction	Effects of Plasticity
Sudden Change			
Single Trait	Change in mean phenotype described by univariate Lande equation (eqn 1). Exponential approach to new optimum (Gomulkiewicz and Holt 1995).	Maximal amount of environmental change the population can handle depends on width of fitness function, intrinsic growth rate, initial population size, and genetic variation (Gomulkiewicz and Holt 1995)	Approach to new optimum is facilitated by temporary increase in phenotypic plasticity and a concomitant release of hidden genetic variation (Lande 2009). Plasticity reduces extinction risk (Chevin and Lande 2010).
Multiple Traits	Change in mean phenotype described by multivariate Lande equation (eqn 3). Trajectory to new optimum biased toward genetic line of least resistance (Schluter 1996). Lag load decreases roughly exponentially (Chevin 2013).	As for single trait. Extinction risk depends on genetic variance in direction of selection (Gomulkiewicz and Houle 2009).	No models available for the context of environmental change.
Gradual Change			
	Population follows the optimum with a constant lag (Lynch and Lande 1993). Trait correlations can induce permanent maladaptation in traits under stabilizing selection (‘flying-kite effect’; Jones et al. 2004). Genetic variance increases (Bürger and Lynch 1995; Bürger 1999).	Critical rate of environmental change (eqn A6) increases with genetic variance in direction of moving optimum and with intrinsic growth rate and is maximal at intermediate strength of selection (Lynch and Lande 1993; Bürger and Lynch 1995; Gomulkiewicz and Houle 2009), see Fig. 3.	Adaptive plasticity reduces the perceived speed of environmental change. ⇒ increases critical rate of change, decreases phenotypic lag, and decreases rate of genetic evolution. Effects may be counteracted by costs of plasticity (Chevin et al. 2010).
Random Change			
	Population's ability to track the optimum increases with autocorrelation of fluctuations (Lande and Shannon 1996; Chevin 2013). Autocorrelated fluctuations increase genetic variance, whereas uncorrelated fluctuations do not (Bürger 1999).	Extinction risk elevated if fluctuations are uncorrelated and occur in directions with strong selection and high genetic variation (Chevin 2013).	Strong plasticity increases extinction risk if environmental cues are unreliable (Reed et al. 2010). Predictable fluctuations can select for increased plasticity.
Spatial Heterogeneity			
	Spatial heterogeneity constrains adaptation. Trait interactions can induce ‘counter-gradient’ clines, causing traits to evolve away from the optimum (Duputié et al. 2012).	Population growth is maximized at intermediate dispersal rates. Critical rate of environmental change increases if spatial selection gradient is aligned with direction of abundant genetic variation and weak stabilizing selection (Duputié et al. 2012).	If plasticity is expressed before (after) migration, it increases (reduces) migration load and can decreases (increase) species ranges. Expressed plasticity increases near range limits (Chevin and Lande 2011; Thibert-Plante and Hendry 2011).

### Adaptation of a single quantitative trait

#### Sudden environmental change

In the sudden-change scenario, a population that is well adapted to its environment is displaced from the fitness peak by a sudden shift of the optimum. Phenotypic evolution is relatively straightforward: the mean phenotype will approach the new optimum exponentially (because the fitness gradient decreases in the vicinity of the optimum) (Lande 1976b). The key question is whether evolution is fast enough in cases where, immediately after the environmental change, the population mean fitness is less than 1. In this case, the population size will initially decline, setting off a ‘race’ between adaptation and extinction. Gomulkiewicz and Holt (1995) showed that evolutionary rescue is possible only if the initial maladaptation after the environmental change is not too large and the initial population size is high.

#### Gradual environmental change

The situation is quite different if the optimum changes gradually rather than suddenly. In the simplest case, the optimum increases linearly at rate *k*. This model has been analyzed by Lynch et al. (1991), Lynch and Lande (1993), and Bürger and Lynch (1995) and later been extended by various authors (see below). An excellent summary is given in the study by Bürger and Lynch (1997). As the behavior of this model is highly instructive, we will describe it in some detail (see also Appendix 1).

Assume again that the original population is well adapted. As the optimum starts moving, selection becomes gradually stronger (see eqn A4). Consequently, the population will initially evolve slowly, and the lag between the optimum and the population mean phenotype will increase (the population ‘slips off’ the fitness peak). However, as the distance to the optimum increases, so does the selection gradient, until finally a state of dynamic equilibrium is reached, at which the rate of evolution exactly matches the rate of environmental change (see Fig. 1A and eqn A5). Whether or not the population survives depends on the mean fitness at this distance from the optimum (i.e., on the lag load, which is approximately proportional to *k*^2^; Lande and Shannon 1996). One can thus calculate a *critical rate of environmental change k*_crit_ (eqn A6), which is the maximal rate of change the population can handle. If the environment changes faster than *k*_crit_, the lag load becomes so large that the population can no longer maintain itself. Extinction usually follows quickly, because the reduction in population size leads to a loss of genetic variation, which further undermines the population's ability to adapt.

Thus, in contrast to the sudden-change scenario, evolutionary rescue in a gradually changing environment requires that the population maintain a positive growth rate at all times. This is a consequence of the ‘relentless’ movement of the optimum, which means that a population that has fallen behind in the race will get no chance to catch up. It also is noteworthy that extinction in this model usually is not due to a lack of genetic variance (except in the final phases of the collapse), nor due the classical ‘cost of selection’ (i.e., the required number of selective deaths, Haldane 1957). Rather, the population dies out because *all* individuals (not just the less adapted ones) have low fitness.

The critical rate of environmental change is directly proportional to the additive genetic variance and the square root of the maximal population growth rate (see eqn A6). The dependence on the width of the fitness landscape – or conversely, the strength of stabilizing selection – is more complex: as shown in the first row of Fig. 3, for constant 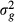, *k*_crit_ is maximal at small to intermediate values of the parameter ‘*V*_*s*_’ which measures the effective width of the fitness function. In other words, the population can support the fastest environmental change if stabilizing selection is strong, but not too strong. The drop-off in *k*_crit_ at low or high values of *V*_*s*_ can be explained by the two kinds of genetic load introduced above. On the one hand, very strong selection (i.e., in a steep and narrow fitness landscape; small *V*_*s*_) induces a high standing load, which reduces the realized growth rate and diminishes the ability of the population to tolerate environmental change. On the other hand, sufficiently weak selection in combination with a moving optimum increases lag load, because the population will follow the optimum at a greater distance. This somewhat counter-intuitive result is due to the fact that, on a flatter fitness landscape, reaching a given selection gradient requires a larger decrease in mean population fitness (see above and Fig. 1). In other words, whereas strong selection keeps the population close to the optimum at high mean fitness, weak selection, precisely because it is ineffective, allows the population to slip farther off the fitness peak. Therefore, weak selection in combination with a constantly moving optimum represents a ‘slippery slope’ that can be very dangerous for population survival (see discussion in Bürger and Lynch 1995 and Huey and Kingsolver 1993). Bürger and Lynch (1995) also showed that the critical rate of change is further decreased by genetic drift of the mean phenotype in small populations and by stochastic fluctuations of the optimum around the linear trend (see also Björklund et al. 2009).

In many quantitative-genetic models, the additive genetic variance 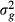 is assumed to be constant. Over short timescales, this may be approximately true, but over longer timescales, 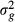 is itself subject to evolutionary change, and it is this fact that makes expressions for *k*_crit_ (such as eqn A6) ‘deceptively simple’ (Bürger and Lynch 1995). Explaining the evolution and maintenance of genetic variation is one of the perennial problems in theoretical population genetics, and no fully satisfactory model has as of yet been found (Barton and Turelli 1989; Bürger 2000; Barton and Keightly 2002; Johnson and Barton 2005; Hill 2010). Before the environmental change, the population may be assumed to be at mutation-selection-drift balance, for which several approximations have been developed (Lande 1976a; Turelli 1984; Bürger 2000; Alvarez-Castro et al. 2009). In the second row of Fig. 3, we follow Bürger and Lynch (1995) by showing the predicted values of *k*_crit_ (in units of the phenotypic standard deviation *σ*_*p*_, see below) when 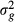 is chosen according to the so-called stochastic house-of-cards approximation (Bürger et al. 1989). Doing so takes into account that populations under weak selection have higher genetic variance, which may offset the negative effects of weak selection on the lag load (see above) and lead to a positive relationship between the width of the fitness landscape and *k*_crit_ (see Huey and Kingsolver 1993). However, this is still not the whole story, because once the optimum starts moving, 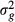 is expected to increase. This increase is mainly due to the rise in frequency of previously rare alleles, and it is strongest in large populations (Bürger 1999): for example, under standard values of mutational and selectional parameters, 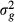 increases up to 4-fold in populations with *N*_*e*_ > 5000. In contrast, selection has little impact on 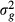 if *N*_*e*_ < 200−300 (Bürger 1999), which might explain why genetic variances usually do not increase in artificial selection experiments, as noted by (Johnson and Barton 2005). A useful upper limit for the genetic variance in small populations (*N*_*e*_ < 500, Bürger and Lynch 1995) is the neutral expectation 2*V*_*m*_N_*e*_, where *V*_*m*_ is the input of genetic variance from new mutations (a typical value is 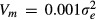, Lande 1976a; Lynch 1988). In summary, evolution of the genetic variance may increase the prospects of population survival, but mostly in large populations. It should be noted, though, that the increase in variance takes time and may come too late for populations subject to strong environmental change.

#### Fluctuating selection

In addition to sudden or gradual changes, most environments are subject to stochastic fluctuations. We have already seen that superimposing fluctuations on a linear trend in the optimal phenotype increases population extinction risk and decreases the critical rate of environmental change *k*_crit_ (Bürger and Lynch 1995). Here, we briefly discuss the effects of fluctuations around a constant mean. Uncorrelated fluctuations (white noise) in the optimal phenotype resemble a sudden-change scenario that is repeated each generation. Such fluctuations can incur strong selection, but the responses of the population will not add up to large changes over longer timescales (Gingerich 1983; Gibbs and Grant 2006). In addition, genetic responses to selection in one generation are likely to be maladaptive in the next generation, and therefore, the lag load will be high (Lande and Shannon 1996; Bürger 1999; Chevin 2013). Consequently, uncorrelated fluctuations do not lead to a significant increase in genetic variance relative to constant stabilizing selection (Bürger 1999). An exception exists, however, if a species possesses dormant stages such as seeds or resting eggs or if generations are overlapping but selection acts only on juveniles. In these cases, the ‘storage effect’ allows the maintenance of genetic polymorphism and, hence, high levels of variation (Chesson and Warner 1981; Hairston et al. 1996). Environmental fluctuations can also select for phenotypic plasticity, provided the state of the environment can be assessed by a reliable cue (Tufto 2000), or for bet-hedging, if there is no such cue (Svardal et al. 2011).

In contrast to uncorrelated fluctuations, autocorrelated fluctuations are more similar to the gradual-change scenario, and a population with sufficient genetic variance can follow the optimum and maintain high fitness (Charlesworth 1993; Lande and Shannon 1996; Chevin 2013). Consequently, autocorrelated fluctuations can lead to significant increases in genetic variation (Bürger 1999).

### Adaptation of multiple correlated traits

When several traits are under selection, the above analyses need to be extended to account for the effects of genetic correlations. As mentioned above, genetic correlations tend to bias the phenotypic response to selection toward the leading eigenvector of the **G**-matrix, **g**_max_ (the ‘genetic line of least resistance’; Schluter 1996). In the sudden-change scenario, an evolving population will still reach the new optimum, although not along the most direct path (Fig. 2A). While the optimum is approached, the lag load decreases as a sum of exponential terms, with rates given by the eigenvalues of the matrix of selection responses (Chevin 2013). Adaptation is fastest and evolutionary rescue is most likely if the angle between the direction of selection and **g**_max_ is small (Gomulkiewicz and Holt 1995; Schluter 1996).

Under gradual environmental change, selection for a moving optimum may cause permanent maladaptation of traits (or trait combinations) that are under pure stabilizing selection (i.e., orthogonal to the direction of the optimum). As illustrated in Fig. 2B, the initial response to selection is biased toward **g**_max_, causing the population to rise above the line of the moving optimum, a phenomenon that has been termed the ‘flying-kite effect’ (Jones et al. 2004). Eventually, the rise comes to a halt, as stabilizing selection in the respective direction increases, and the population's trajectory continues in parallel to that of the optimum. Again, population survival will depend on the lag load at this steady state. A critical rate of environmental change can be calculated in analogy to the univariate case (see Appendix 2). It depends not only on the shape of the fitness landscape, but also on the direction of the optimum and the structure of the **G**-matrix. In particular, the critical rate is high if the optimum moves in parallel to **g**_max_, and it is lowest if the optimum moves in a direction of low genetic variation (see Hellmann and Pineda-Krch 2007 for graphical illustrations and a discussion of the consequences for conservation biology).

As in the univariate case, many studies assume that the **G**-matrix is roughly constant over the timescale of interest. Evolution of the **G**-matrix has been studied in a recent series of papers by Jones, Arnold and Bürger (Jones et al. 2003, 2004, 2007, 2012; for review see Arnold et al. 2008). In accordance with previous studies (Barton and Turelli 1987; Bürger and Lynch 1995; Jones et al. 2004), Jones et al. (2012) found that, irrespective of the mode of environmental change (gradual, episodic, stochastic), genetic variance increases in the direction of environmental change. While this facilitates the response to selection, the phenotypic lag also induces a skew in the distribution of breeding values (unfit phenotypes ‘trailing behind’), which restrains the response to selection. Generally, the two phenomena do not offset each other (Jones et al. 2012), requiring inspection for every individual case. These results highlight the need for caution when iterating the Lande equation or interpreting **G**'s eigenvalues (Kirkpatrick 2009). Under pure stabilizing selection, the **G**-matrix tends to align itself with the fitness landscape, that is, genetic variance is highest in directions with weak selection. **G** depends, however, also on the distribution of new mutations, that is, the **M**-matrix (Jones, et al. 2003, 2007), and on gene flow (Guillaume and Whitlock 2007; Franks et al. 2014).

### Genetic basis of adaptation

The quantitative-genetic models we have considered so far are most accurate if adaptation is based on a large number of loci with small individual effects. In this section, we briefly discuss several issues that arise when this assumption is relaxed.

The first question is how the rate of adaptation is affected by alleles of large effect. If the same total progress toward the optimum can be made by the fixation of either a single allele of large effect or many alleles with small effects, adaptation will be faster in the former case, because selection on the large alleles is more effective (Gomulkiewicz et al. 2010; for the same result in a different context, see also Gavrilets et al. 2007; Rettelbach et al. 2011). In Appendix 3, we calculate the rate of phenotypic evolution due to the fixation of a major allele and show that it can be quite high, at least while the allele is at intermediate frequency. For quantitative traits that are determined by a combination of small- and large-effect loci, Gomulkiewicz et al. (2010) showed that adaptation is fastest when both classes of loci are evolving. For the same situation, Chevin and Hospital (2008) demonstrated that ‘background’-adaptation from minor loci, by successively reducing the selective advantage of a large-effect allele, can significantly affect its trajectory, and even prevent fixation. The exact outcome crucially depends on the initial allele frequency, the distance from the optimum, and the amount of genetic variation provided by the minor loci.

Another question is, however, how likely beneficial alleles with large effect are in the first place. In a multivariate context, Fisher (1930) used his classical ‘geometric model’ to argue that alleles (i.e., mutations) with large effect that pleiotropically affect multiple traits are most likely to be deleterious. As pointed out by Kimura (1983), however, Fisher neglected the fact that, among beneficial mutations, the few mutations with large effect have a higher fixation probability than the more common mutations with small effects. In the last two decades, numerous theoretical studies have developed predictions for the distribution of phenotypic and fitness effects of both new and fixed mutations (e.g., Martin and Lenormand 2006a, 2008; Keightley and Eyre-Walker 2007; Yeaman and Whitlock 2011), and many models have concluded that the role of mutations with major effects in adaptation is surprisingly large (reviewed by Orr 2005). However, almost all of these models have considered a sudden-change scenario. Under gradual environmental change, results might be very different. In particular, Collins et al. (2007) and Kopp and Hermisson (2007, 2009a,b) showed that a slowly moving optimum favors adaptation by small mutations.

Finally, many authors have studied adaptation and evolutionary rescue from a single large mutation. As these models usually do not refer to quantitative traits, we only point out the relevant literature: for the probability of evolutionary rescue, see Gomulkiewicz and Holt (1995); Holt and Gomulkiewicz (1997); Orr and Unckless (2008); Uecker and Hermisson (2011); Martin et al. (2013); for the fixation probability of a new mutation in a changing environment, see Uecker and Hermisson (2011); Kirkpatrick and Peischl (2013); Martin et al. (2013); and for the probability of adaptation from standing genetic variation versus new mutations, see Hermisson and Pennings (2005); Martin et al. (2013).

### Maximal sustainable rates of evolution?

**Figure A1:**
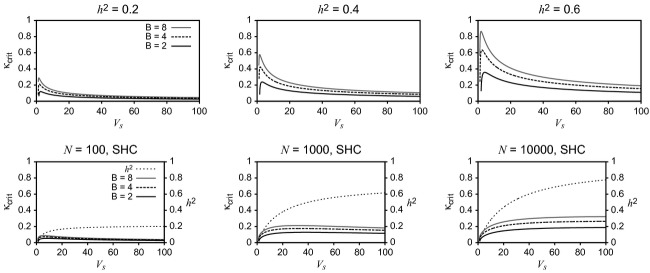
The critical rate of phenotypic evolution, *κ*_crit_ = *k*_crit_ / *σ*_*p*_ (eqn A7) expressed in *haldanes*, for the one-dimensional moving-optimum model (A2) (after Bürger and Lynch 1995), as a function of the width of the fitness function 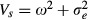 (with 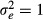), for various values of the reproductive potential *B*. The top row shows results for three different values of heritability 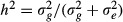. In the bottom row, 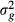 has been set to the value predicted by the stochastic house-of-cards (SHC) approximation under pure stabilizing selection for three values of the population size *N*. The SHC approximation is given by 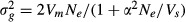 (Bürger and Lynch 1995), where *V*_*m*_ is the mutational variance, *α*^2^ is the variance of the effect of new mutations, and *N*_*e*_ ≈ 2*BN* / (2*B*−1) (Bürger and Lynch 1995) is the effective population size. The figures are for *V*_*m*_ = 0.001 and *α*^2^ = 0.05. The thin dotted line gives the heritability *h*^2^ associated with 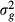 (SHC).

A well-known prediction from the models by Lynch and Lande (1993) and Bürger and Lynch (1995) is that of a ‘maximal sustainable rate of evolutionary change’ on the order of 0.1 *haldanes* or less. This value is simply a ballpark estimate of the critical rate of gradual environmental change, *k*_crit_ (eqn A6), scaled by the phenotypic standard deviation and parametrized with realistic parameter values (see Appendix 1 and Fig. A1). As, at the dynamic equilibrium, the population follows the optimum with a constant lag, the rates of environmental and phenotypic change are ‘formally equivalent’ (Bürger and Lynch 1995). For clarity, we will denote the rate of phenotypic change in *haldanes* by *κ*_crit_ = *k*_crit_/*σ*_*p*_ (eqn A7).

Barrett and Hendry (2012) note that it is ‘tempting’ to use *κ*_crit_ = 0.1 as a benchmark for empirically observed evolutionary rates, the idea being that rates near or above this value might be cause for concern because they are not ‘sustainable’ (see also Hendry and Kinnison 1999). Based on earlier meta-analysis (Hendry and Kinnison 1999; Kinnison and Hendry 2001; Hendry et al. 2008), these authors conclude that most rates of change are below 0.1. In contrast, Gingerich (2009) argued that evolutionary rates on the order of 0.1 and 0.3 *haldanes* are common, but his analysis relied on an interpolation technique (log-rate-log-interval plots) that is sensitive to measurement error when real rates of change are small (Hunt 2012 and below). Barrett and Hendry (2012) also warn, however, that theory-derived critical rates rely on ‘many unrealistic assumptions, such as perpetual persistence under constant environmental change’ and that ‘critical rates for natural populations over time frames of conservation interest could be very different’.

There are several points to be made here (see also Appendix 4). First, and obviously, a universal *κ*_crit_ of 0.1 *haldanes* cannot be more than a rule of thumb. Critical rates may be higher under strong selection, high heritabilities and in large populations (Fig. 3). Second, some of the reasons for population extinction found by Bürger and Lynch (1995) – such as random but autocorrelated fluctuations in genetic variance – are, indeed, mainly a long-term concern under sustained environmental change. Third, however, the critical rate in eqns (A6) and (A7) is simply equivalent to the (instantaneous) rate of evolutionary change that can be achieved without a decrease in population size, as a function of (i) the genetic variance, (ii) the reproductive capacity of the population, and (iii) the shape of the fitness landscape (see discussion of mean fitness vs. fitness gradient above). Faster evolution is possible temporarily, but only at the cost of a reduction in population size. To quantify this effect, in Appendix 4, we estimate maximal rates of environmental and phenotypic change when allowing modest population decline over a limited time frame (e.g., the population is to maintain a minimal size of 50 individuals for 50 generations). As shown in Fig. 4, this provision leads to modest increases in *κ*_crit_ in large populations (typically around 30%), whereas the effect in small populations is negligible (in particular, in the light of the stochastic variations discussed below). In summary, *κ*_crit_ is, indeed, likely to often be around or below 0.1 *haldanes*. Faster observed rates may be a sign that the population is under stress (e.g., the well-known example of Darwin's finches during a drought, where beak-size increased by 0.66 standard deviations, but 85% of the population died; Grant and Grant 2006) or may indicate that part of phenotypic change is due to plasticity (see below). Temporarily high rates of change may also be achieved by the fixation of a large-effect mutation (Appendix 3).

Small maximal rates of phenotypic change also raise statistical issues (Appendix 5; see also Hendry and Kinnison 1999): detecting a difference of 0.1 standard deviations between two populations requires very large sample sizes (e.g., almost 800 per population for 50% power in a two-sample *t*-test with *α* = 0.05). On the other hand, differences of this magnitude can easily be created by sampling effects (Figs 5, 6; Kinnison and Hendry 2001; Hunt 2012). Indeed, the mean absolute differences in units of phenotypic standard deviations between two samples of size *n* drawn from the same population is 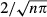 (Hunt 2012), which equals 0.113 for *n* = 100. In finite populations, similar effects occur due to genetic drift and environmental variance (even if the whole population is sampled). The variance of the mean phenotype due to genetic drift is 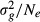, with *N*_*e*_ being the effective population size (Lande 1976a). By a calculation analogous to the one in Hunt (2012), the mean generation-to-generation rate in *haldanes* due to drift is 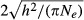, which is 0.025 for *N*_*e*_ = 1000 and *h*^2^ = 0.5. Similarly, the contribution of environmental variance (i.e., genotype-independent random variation in individual phenotypes) to the mean rate of phenotypic change is 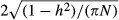 (with *N* being the census population size). Together, these two sources of variation may dominate the generation-to-generation changes in the mean phenotype of small populations (Appendix 5, Figs A6, A7). In summary, maximal sustainable rates of evolutionary change might often be of the same order than various sources of stochastic noise, something which should be kept in mind when interpreting evolutionary rates measured over short timescales.

## The role of phenotypic plasticity

So far, we have only considered genetic adaptation. However, many observed responses to climate change are likely to be plastic (Gienapp et al. 2008; Hendry et al. 2008; Merilä 2012), and assessing the relative importance of plastic and genetic changes is precisely the aim of this special issue of *Evolutionary Applications*. Yet, in its basic form, the question is empirical and cannot be answered by theory alone. While quantitative genetic models can make some tentative predictions about the maximal rates of genetically based evolution (see above), it seems impossible to make general statements about the range and scope of plasticity. Here, we will instead focus on reviewing models that investigate the interaction between plasticity, population dynamics, and genetic evolution. Because several important aspects have already been reviewed elsewhere (Ghalambor et al. 2007; Chevin et al. 2013), our treatment can be short.

Ecological models have investigated the effect of plasticity on population stability and extinction risk in the absence of evolution. Community models including so-called trait-mediated indirect effects (Werner and Peacor 2003) frequently find that phenotypic plasticity mediated by species interactions (e.g., inducible defenses against predators; Tollrian and Harvell 1999) can stabilize population dynamics, even though such a stabilizing influence is not universal (Kopp and Gabriel 2006). If plasticity increases the range of conditions under which a community is stable, it reduces the risk of species extinctions after an arbitrary environmental change (‘plastic rescue’; Kovach-Orr and Fussmann 2013).

For a single population, Reed et al. (2010) studied the impact of phenotypic plasticity on population extinction risk in a randomly fluctuating environment. They found that adaptive plasticity decreases extinction risk, unless the magnitude of plastic responses exceeds an optimal level set by cue reliability (strong responses to unreliable cues tend to be harmful). Chevin et al. (2010) included phenotypic plasticity into the moving-optimum model of Lynch and Lande (1993) and Bürger and Lynch (1995). Assuming a linear reaction norm with slope less than one, plasticity essentially reduces the speed of environmental change perceived by the population. Plasticity thus increases the critical rate of environmental change *k*_crit_ that separates population survival from extinction. In consequence, it increases the maximal rate of phenotypic change, while simultaneously decreasing the rate of genetic evolution. This effect may be reversed at high levels of plasticity if plasticity itself is costly (and hence, reduces the mean fitness of the population).

Gienapp et al. (2013) recently applied both the Chevin et al. (2010) and the Bürger and Lynch (1995) model to anticipate evolution of egg-laying dates in great tits from a well-studied Dutch population. Egg-laying date in this species is a phenotypically plastic trait that depends on spring temperature and is selected to coincide with the peak in caterpillar abundance. Using various modeling techniques, the authors show that, despite plasticity, global warming will create a mismatch between the optimal and realized egg-laying dates, which might threaten population persistence unless it can be closed by genetic evolution. By focusing on the predicted mismatch, the authors were able to parametrize the Bürger and Lynch (1995) model (i.e., eqn A6), even though this model was not built to deal with plasticity. They conclude that, even under a mild climate-change scenario, the predicted rate of environmental change (from the point of view of the population) is close to the theoretical maximal sustainable rate. To parametrize the Chevin et al. (2010) model, Gienapp et al. (2013) assumed that both optimal and realized egg-laying dates correlate with mean spring temperature (measured between mid-March and mid-April). Although the Chevin et al. (2010) model seems to be more suitable for the analysis of a plastic trait, its results are less plausible than those obtained from the Bürger and Lynch (1995) model. In particular, the model predicts that population survival will be facilitated by fast environmental change. The authors argue that this counterintuitive prediction is an artifact, which arises because, with faster temperature increase, mean spring temperature becomes less and less correlated with the true causal variable determining optimal egg-laying date. This highlights the general problem that, frequently, the variables we can measure are just proxys for one or more causal factors. If the proxy is bad, any model will perform poorly. Despite these issues, the study by Gienapp et al. (2013) is exemplary in its combined use of long-term empirical data, climate-change predictions, and models for future optimal and realized behavior.

In the following, we briefly review models in which plasticity can itself evolve. Conditions for the evolution of plasticity are fairly well understood. Plasticity is adaptive if individuals encounter different environmental conditions that favor different phenotypes and that can be assessed by a reliable cue (e.g., Tollrian and Harvell 1999; Ghalambor et al. 2007). Its evolution may be limited by functional constraints, unreliable cues (Tufto 2000) and costs for the necessary sensory and developmental machinery (DeWitt et al. 1998; van Buskirk and Steiner 2009). More recently, however, phenotypic plasticity has been advocated as not only a product, but also a driver of genetic evolution (West-Eberhard 2003; for recent reviews, see Ghalambor et al. 2007; Pfennig et al. 2010; Wennersten and Forsman 2012; Wund 2012). The basic idea is that new phenotypes first appear as a result of environmental induction and only later are fixed via ‘genetic assimilation’ or ‘genetic accommodation’. Here, genetic assimilation corresponds to a loss of plasticity, such that expression of the phenotype becomes independent of environment cues. Genetic accommodation is a more general ‘fine-tuning’ of the novel phenotype via changes in allele frequencies, potentially facilitated by a release of hidden genetic variation (Hermisson and Wagner 2004; Moczek 2007; for more conceptual discussion, see West-Eberhard 2005; Crispo 2007; Ghalambor et al. 2007). The more ambitious versions of this hypothesis – that environmental induction can be at the basis of ‘evolutionary novelties’ (West-Eberhard 2003; Pigliucci et al. 2006; Uller and Helanterä 2011) – appears unaccessible to classical population-genetics modeling. Here, we focus on the less far-reaching question of the role of plasticity in the evolution of existing quantitative traits.

Phenotypic plasticity has traditionally been viewed as delaying genetic evolution. This is certainly true if plasticity is sufficient to ensure continued high fitness of a population in a changing environment. However, there are other scenarios in which plasticity may, indeed, speed up or facilitate genetic change. A simple case is the Baldwin effect (Baldwin 1896; Crispo 2007), where plasticity (specifically, learning) allows a population to survive in a new or changed environment, thereby enabling future genetic adaptation (for models, see Ancel 1999; Pál and Miklós 1999; Ancel 2000; Paenke et al. 2007). Furthermore, plasticity can influence the course of evolution by bringing a population into the domain of attraction of a specific adaptive peak. The probability of a peak shift is highest if plasticity is of intermediate strength (Price et al. 2003). Both mechanisms may play a role in biological invasions as well as adaptation to climate change.

Recently, Lande (2009) proposed a simple model for the role of plasticity in adaptation to an abrupt environmental shift. He considered the evolution of a quantitative trait that is determined by linear reaction norms. That is, for each individual, the trait value is a linear function of an environmental variable, with genetic variation in the slope and intercept of this function (see also Gavrilets and Scheiner 1993). Under the original conditions, a modest level of plasticity (i.e., an intermediate slope of the reaction norm) is favored in a slightly fluctuating environment with constant mean and imperfect cues. At this stage, reaction-norm slope varies between individuals, but the mean phenotype is relatively homogeneous (canalization). When the mean environment changes, genetic variance is increased due to differential plastic responses (decanalization), and selection favors individuals with steep reaction norms, which can best adjust to the new conditions. That is, the population evolves toward the new optimum via the evolution of increased plasticity, allowing high rates of phenotypic change. Subsequently, the reaction norm intercepts increase and slopes decrease, again reaching the optimal degree of plasticity in the new environment (genetic assimilation). Chevin and Lande (2010) added population dynamics to this model and showed that evolving plasticity strongly increases the probability of evolutionary rescue after a sudden environmental change.

## What determines adaptive potential?

Ideally, we would like to be able to predict which species have the potential to adapt to rapid climate change (Williams et al. 2008; Huey et al. 2012). Obviously, phenotypic plasticity will help (see above), but theory can say little more than that. With regard to genetic adaptation, the adaptive potential depends most directly on the genetic variation that is available in the direction of selection. In addition, we may also ask what kind of genetic architectures and evolutionary histories facilitate rapid adaptation. We will discuss these two issues in turn.

### Genetic variance and genetic constraints

For single traits, a short-term measure of adaptive potential is given by the additive genetic variance (see eqn 1), and a lack of such variance corresponds to a genetic constraint (i.e., adaptive potential and genetic constraints are two sides of the same coin). An absolute constraint is present if genetic variance is zero, and a relative constraint if it is low. Gomulkiewicz and Houle (2009) pointed out that if adaptation is too slow to avoid extinction, a relative (or quantitative) constraint is effectively transformed into an absolute constraint. They coined the term ‘demographic constraint’ to refer to this situation and calculated ‘critical amounts of genetic variance’ and ‘critical heritabilities’ that are necessary to prevent extinction under scenarios of sudden and gradual environmental change.

In the multivariate case, an additional source of genetic constraints may arise from genetic correlations. Indeed, even if every single trait has positive genetic variance, the variance for certain trait combinations may be zero (Dickerson 1955). In this case, the **G**-matrix is singular (Lande 1979), that is, at least one of its eigenvectors has a zero eigenvalue. If the selection gradient is parallel to such an eigenvector, it will produce no effect. Regardless of the direction of selection, evolution will be possible only in a lower-dimensional subspace of the original phenotype space (e.g., along a line in two dimensions or a plane in three dimensions). A singular **G**-matrix might be an extreme case (and is difficult to infer statistically). However, relative constraints arise in the same way, whenever an eigenvalue is positive but small. Using their concept of demographic constraints, Gomulkiewicz and Houle (2009) calculated critical values for the smallest eigenvalue of **G** in the worst-case scenario that selection acts exactly in the direction of the corresponding eigenvector.

What is the overall role of genetic correlations in constraining the rate of adaptation? – Walsh and Blows (2009) argued that strong multivariate constraints (weak variation in the direction of selection) might, indeed, be common and could explain the frequent observation of slow evolutionary change despite strong selection on (individually) variable traits. To quantify the distribution of genetic variation, Kirkpatrick (2009) defined a measure of ‘effective dimensionality’


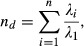
(5)

where the *λ*_*i*_ denote the eigenvalues of the **G**-matrix ordered from the largest (*λ*_1_) to the smallest (*λ*_*n*_). If genetic variation is uniformly distributed among the eigenvectors, *n*_*d*_ takes it maximal value of *n*, whereas it is minimal (equal to 1) when genetic variation is only present along a single axis. A review of empirical estimates of *n*_*d*_ suggests that it is often (much) smaller than the number of traits considered (Kirkpatrick 2009). Thus, genetic variation seems to be concentrated around a few dimensions, meaning that the ability of populations to respond to arbitrary selection pressures may be severely reduced.

However, an alternative approach by Agrawal and Stinchcombe (2009) yields more nuanced results. These authors proposed to compare the increase in mean fitness in response to a given selection gradient for the full **G**-matrix with the expected response when assuming a (hypothetical) modified **G**-matrix in which all off-diagonal entries (i.e., all covariances) have been set to zero. Using data from empirical estimates of **G**- (or **P**)-matrices and selection gradients, they found that removing genetic correlations sometimes increases and sometimes decreases the rate of adaptation and that often, the effect is minor. In this context, it is worth pointing out that genetic correlations do not necessarily decrease the variance in a particular direction. For example, adding arbitrary covariances to a diagonal **G**-matrix can only increase genetic variation in the direction of the leading eigenvector (Horn and Johnson 1985, p. 194).

Theoretical studies have used two approaches to quantify genetic constraints (for a review of measures, see Walsh and Blows 2009). If the selection gradient is known, adaptability and constraints should be expressed relative to its direction. A sophisticated set of measures was proposed by Hansen and Houle (2008), who distinguish ‘respondability’ (the magnitude of overall phenotypic change in response to selection in a given direction with unit magnitude), ‘evolvability’ (the magnitude of response in the direction of selection), ‘conditional evolvability’ (the magnitude of response in selected traits if correlated traits are forced to remain constant), and ‘autonomy’ (the fraction of genetic variation in a trait that is independent of potentially constraining characters). For cases where the direction of selection is not known, several authors have calculated mean rates of adaptation over a distribution of possible selection gradients (Hansen and Houle 2008; Kirkpatrick 2009; Chevin 2013). When the distribution of selection gradients is uniform, genetic correlations have no effect on the mean rate of adaptation, because high rates in directions of large variation are offset by low rates in directions of small variation (Hansen and Houle 2008; Kirkpatrick 2009). When the distribution of gradients is not uniform, however, the mean rate of adaptation is highest if selection gradients tend to coincide with directions of large genetic variation (Chevin 2013).

### Other determinants of adaptive potential

We now go on to discuss a broader view of adaptive potential and evolvability. Sexual reproduction and genetic recombination have long been hypothesized to facilitate adaptation to changing environments (e.g., by bringing together alleles on the same genome and reducing the effect of clonal interference). For a gradual-change model, this was confirmed via simulation by Bürger (1999) (see also Charlesworth 1993; Waxman and Peck 1999). In particular, the increase in genetic variance under directional selection (see above) is almost absent in asexual populations.

Several theoretical studies have compared adaptation in (sexual) haploid and diploid populations, but the results are complex. Haploid populations can be expected to evolve faster than diploid populations, because selection is more efficient in haploids (Orr and Otto 1994; Otto and Gerstein 2008), and this was confirmed experimentally in yeast (Gerstein et al. 2011). Nevertheless, haploid populations were invaded by diploid strains (Gerstein and Otto 2011). While in this case, the ‘cryptic fitness advantage’ was attributed to negative frequency-dependent selection, a more general advantage to diploidy was proposed by Sellis et al. (2011). Using the framework of Fisher's geometric model, these authors argued that heterozygote advantage is a natural consequence of adaptation in diploids, at least in populations that are close to a phenotypic optimum. (The reason is that mutations often have smaller phenotypic effects in heterozygotes than in homozygotes, such that heterozygotes may have a fitness advantage, while homozygotes already overshoot the optimum – a probability that increases with the number of phenotypic dimensions.) As heterozygote advantage favors the maintenance of polymorphism, diploids are expected to have higher levels of genetic variation, conferring them an increased adaptive potential in case of rapid environmental change. Indeed, simulations showed that, in fluctuating environments, diploid populations maintained higher mean fitness than haploids, despite a larger standing load (Sellis et al. 2011).

Again using Fisher's geometric model, Orr (2000) argued that evolvability is reduced in complex organisms, because mutations are more likely to have negative pleiotropic side effects. This ‘cost of complexity’ can, however, be reduced by a modular organization (Welch and Waxman 2003). Indeed, several studies have concluded that ‘effective complexity’ is low in many organisms (e.g., Martin and Lenormand 2006b; Lourenco et al. 2011). Such low dimensionality/pleiotropy is predicted to increase the proportion of beneficial mutations with large effect, which in turn can facilitate adaptation (Gomulkiewicz et al. 2010). Gene-network models also predict that small network size leads to an increased rate of adaptation, faster population recovery and higher critical rates of environmental change (Malcom, 2011a, b). Along similar lines, mutational robustness (i.e., the probability for genotypes connected by mutations to express the same phenotype) can paradoxically increase the adaptive potential of a population by allowing synonymous genetic variants to accumulate, thus increasing the mutational neighborhood of a given phenotype (Gavrilets 1997; Fontana and Schuster 1998; Wagner 2008; Draghi et al. 2010).

Finally, adaptive potential is likely to be influenced by a species' evolutionary history. In particular, species that have evolved in variable environments are more likely to survive future environmental change than species that have long lived under very constant conditions. The idea is not only that past fluctuations endow a species with increased genetic variation (see above), which has been pretested by selection in past environments (Masel 2006; Wagner 2007; Hayden et al. 2011), different habitats, or even in another species (e.g., introgression) (Rieseberg et al. 2003; Barrett and Schluter 2008), but also that the species may have evolved increased plasticity and a more flexible genetic architecture (Hansen 2006). Indeed, the last two points might be related. Several recent models have shown that genetic networks that evolved to express plasticity also allow for faster genetic adaptation (Espinosa-Soto et al. 2011; Fierst 2011; Draghi and Whitlock 2012).

On the other hand, species that have evolved under highly stable conditions are expected to be the most sensitive to environmental change (Overgaard et al. 2011). In particular, there is concern that tropical ectoterms might be unable to resist increasing temperatures (Janzen 1967; Ghalambor et al. 2006; Deutsch et al. 2008; McCain 2009; Hoffmann et al. 2012; Urban et al. 2014). Indeed, such species are characterized by narrow thermal tolerance curves (Amarasekare and Savage 2012) and have narrow altitudinal ranges (McCain 2009). If genetic variation in the optimal temperature is proportional to the width of the thermal tolerance curve (as has been demonstrated for *Drosophila*; Kellermann et al. 2009; Schilthuizen and Kellermann 2014), they should also have reduced critical rates of environmental change (Huey and Kingsolver 1993). Quantitative predictions about extinction risk are difficult, however, because most studies on thermal tolerances provide only relative, not absolute, fitnesses (Deutsch et al. 2008; Martin and Huey 2008; Bonebrake and Mastrandrea 2010).

## Adaptation in space

Real populations are distributed in space, and they can react to environmental change by migration in addition to genetic evolution and plasticity (Parmesan 2006; Schloss et al. 2012). Here, we are not primarily interested in range shifts, but instead focus on the effects of gene flow on local adaptation in changing environments.

A natural extension of the gradual-change model discussed above considers a shifting environmental gradient, that is, an optimum that changes in both space and time. Building on earlier models by Pease et al. (1989), Kirkpatrick and Barton (1997) and Polechová et al. (2009), Duputié et al. (2012) recently investigated adaptation of multiple quantitative traits in response to such a shifting gradient. In particular, they addressed how multivariate genetic constraints and gene flow alter the adaptive potential. While gene flow from maladapted populations can potentially constrain local adaptation, it may also promote population persistence by enabling the exploitation of larger geographic ranges and by spreading favorable alleles (Schiffers et al. 2013). Consequently, regardless of the number of traits under selection, the critical rate of environmental change is maximized when dispersal is neither too weak nor too strong (Alleaume-Benharira et al. 2006; Duputié et al. 2012). Population persistence also strongly depends on the slope of the spatial gradient. When the gradient is weak (i.e., the loss of fitness per unit space is small), the population remains well adapted over a wide range. Conversely, a steep gradient constrains the range. In this case, population persistence depends heavily on the geometric relation of the **G**-matrix, the shape of the fitness landscape and the direction of the spatial gradient. In particular, adaptive constraints are minimal whenever the spatial gradient is collinear with the direction of weakest stabilizing selection and largest genetic variance. Similar to the ‘flying-kite effect’ (Jones et al. 2004), Duputié et al. (2012) also found that, when there is indirect selection on negatively correlated traits, adaptation in one trait can cause another trait to develop a spatial gradient in the direction opposite to its optimum. When genetic variances are allowed to evolve (as a consequence of selection and gene flow, see above), univariate models have shown that sufficiently large populations can be perfectly adapted over their whole range, albeit at the cost of an increased standing load (Barton 2001; Polechová et al. 2009; Bridle et al. 2010).

The effect of gene flow on the **G**-matrix has been studied by Guillaume and Whitlock (2007). Using a continent-island model, these authors showed that a migration rate of about one individual per generation increases the size of **G** by up to 3-fold and may cause its shape and orientation to ‘flip’ (albeit only over timescales of several hundred generations). These effects are particularly pronounced if other factors acting on **G**, such as the input of mutational variance and mutational or selective correlations, are weak.

The effect of phenotypic plasticity on local adaptation and the colonization of new habitats has been studied by Chevin and Lande (2011) and Thibert-Plante and Hendry (2011). Both studies found that plasticity can facilitate colonization of new habitats, especially if it is expressed after migration (i.e., juvenile dispersal). However, no studies to date have considered the joint effect of plasticity and genetic adaptation in spatially explicit models under environmental change.

## Beyond single species

Real populations do not evolve in isolation but are embedded in a network of ecological interactions, and so predictions of responses to climate change should be made in a community context. Several studies have investigated the effects of interspecific competition on the rate of adaptation and the likelihood of evolutionary rescue. Both positive and negative effects are possible. The presence of competitors can reduce the rate of adaptation in a focal species by reducing its population size (and, hence, genetic variance or mutational input) and by ‘blocking’ the access to new ecological niches (Johansson 2007; Jones 2008; Jones and Gomulkiewicz 2012; Osmond and Mazancourt 2013). This effect increases the lag load, decreases the critical rate of environmental change and can contribute to species extinctions. On the other hand, competition may also facilitate adaptation if a competitor (or predator) ‘pushes’ a focal species in the direction of the new optimum (Jones 2008; Osmond and Mazancourt 2013). Osmond and Mazancourt (2013) argue that both effects can be found in recent studies of character displacement in Darwin's finches (Grant and Grant 2006). Evolution may also be sped up by competitive release if climate change causes a competitor to go extinct (Poloczanka et al. 2008).

In the presence of a shifting spatial gradient (see above), community evolution depends on the interaction of local adaptation and dispersal (de Mazancourt et al. 2008; Urban, et al. 2012a, b). De Mazancourt et al. (2008) used simulations of a multipatch model to show that species often shift their range to new habitats rather than adapting to their altered current habitat and that this effect is stronger in species-rich communities. Urban et al. (2012a) use the term ‘competitive constraint’ to describe the situation where a local species is prevented from adapting to a changing environment because its habitat is being invaded by a competing species already adapted to the new conditions. This effect is a possible explanation for niche conservatism (Wiens et al. 2010) during contemporary evolution. The opposite effect is also possible; however, local adaptation of a resident species can prevent the establishment of a later-arriving invader (monopolization effect, Urban and de Meester 2009; Urban et al. 2012a). And even mal-adapted residents can slow range expansions of dispersing species into newly available habitats (‘boxcar effect’: species can climb climate gradients only as fast as species further up the line; Urban et al. 2012b). In summary, predicting community response to environmental change requires considering the interactions of two local processes (local community dynamics; local adaptation) and two regional processes (immigration from regional species pool and immigration from regional genotype pool, Urban et al. 2012a).

If we move beyond pairwise interactions, both rapid evolution and phenotypic plasticity have been shown to contribute to community stability (e.g., Werner and Peacor 2003; Yamamichi et al. 2011). Kovach-Orr and Fussmann (2013) coined the terms ‘evolutionary and plastic rescue’ to describe situations where this enhanced stability prevents species extinctions after an environmental change. Finally, evolutionary responses to climate change in complex communities will not always increase the chances in population survival, but may instead lead to ‘evolutionary suicide’ (Ferriére and Legendre 2013).

## Conclusions

We have reviewed theoretical models of adaptation to changing environments, with a focus on evolutionary rates of quantitative traits. Unlike models of evolutionary rescue by single mutations, the majority of quantitative-genetic models consider gradual rather than abrupt environmental change. Early models for single traits have introduced the concept of a critical rate of environmental change or maximal sustainable rate of evolution, beyond which long-term persistence is not possible (Lynch and Lande 1993; Bürger and Lynch 1995). Subsequently, this concept has been extended to include multivariate selection (Gomulkiewicz and Houle 2009), spatial variation (Duputié et al. 2012) and phenotypic plasticity (Chevin et al. 2010). Despite the added complexity, it seems unlikely that genetic evolution can frequently produce rates of change beyond 0.1 *haldanes* for more than a few generations. Higher observed rates are thus likely to be due to phenotypic plasticity, or to be accompanied by population decline. Empirical tests of this theory are challenging (Gomulkiewicz and Shaw 2013), in part due to a strong impact of nonselective stochastic factors on observed evolutionary rates, and only one study (Gienapp et al. 2013) has attempted to estimate the critical rate of change for a natural population (for estimates based on physiological models and laboratory data, see Huey and Kingsolver 1993 and Willi and Hoffman 2008). We hope that, in the future, more such estimates will become available from well-studied populations. Another promising avenue is experimental evolution under gradually changing conditions (Collins 2004; Perron et al. 2008; Lindsey et al. 2013).

We have also identified four developing areas that significantly increase the realism of the basic models. These include the interactions between phenotypic plasticity and genetic evolution, the role of genetic architecture for the adaptive potential, adaptation to shifting spatial gradients and the influence of interspecific interactions on rates of adaptation. The former two concern mainly internal (organismal) features, whereas the latter two are about external (environmental) factors. Further integrating these various models promises to significantly advance our understanding of species adaptations to climate change.

## Figures and Tables

**Figure A2:**
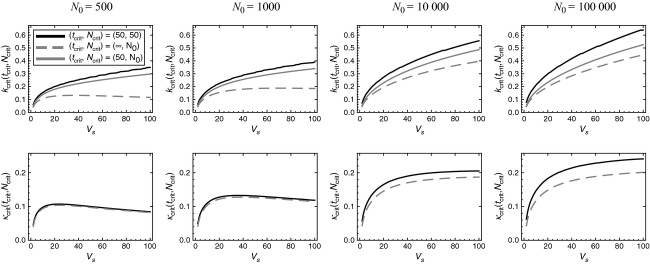
Critical rates of environmental change *k*_crit_ (top row) and the corresponding rates of phenotypic evolution *κ*_crit_ (bottom row), under the premise that the population maintains a minimal size of *N*_crit_ individuals over *t*_crit_ generations. The case *t*_crit_ = ∞, *N*_crit_ = *N*_0_ (where *N*_0_ is the initial population size, which equals the carrying capacity; dashed line) corresponds to the case investigated by Bürger and Lynch (1995). The case *t*_crit_ = 50,*N*_crit_ = *N*_0_ (gray line) is given by eqn A14. In the bottom row, solid and dashed gray lines are identical, because *κ*_crit_ (*t*_crit_, *N*_0_) = *κ*_crit_ (∞, *N*_0_). Parameters are as in the bottom row of Fig. A1 with *B* = 2.

**Figure A3:**
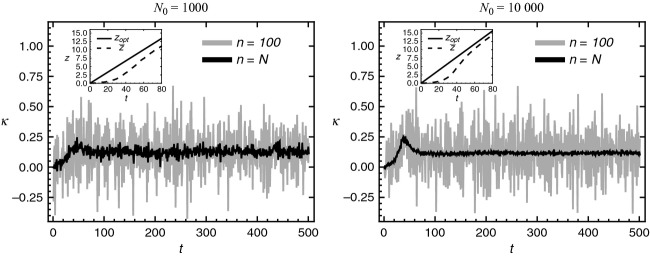
Observed generation-to-generation rates of phenotypic change *κ* in *haldanes* for the entire population (*n* = *N*, black line) or based on a sample of *n* = 100 individuals (gray line), for two simulation runs with carrying capacities (and initial population sizes) *N*_0_ = 1000 and *N*_0_ = 10000, respectively, and parameters as in Fig. A2. The inset shows the trajectories of the mean phenotype 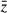 and the phenotypic optimum *z*_opt_. The spike in *κ* around generation 40, which partially closes the large initial phenotypic lag (insert), is due to an increase in genetic variance (see main text). Fluctuations in the black line reflect genetic drift and environmental variance, whereas those in the gray line are largely due to sampling effects. In addition, rates measured in *haldanes* vary due to fluctuations in the phenotypic variance 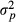 (for potential problems of scale, see Hereford et al. 2004; Hansen and Houle 2008).

**Figure A4:**
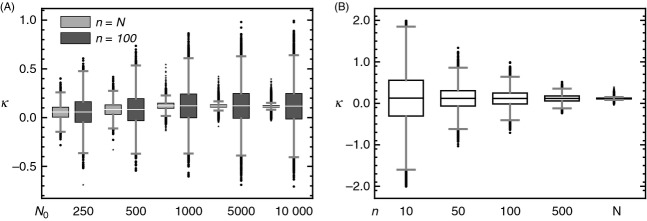
Distribution of observed generation-to-generation rates of phenotypic change *κ* in *haldanes*, over 100 simulation runs similar to those in Fig. A3. In (A), rates based on the entire population (*n* = *N*) or on samples of size *n* = 100 are shown for various initial population sizes (and carrying capacities) *N*_0_. In (B), *N*_0_ = 10000 was kept constant and only sample size *n* was varied. Other parameters are as in Fig. A3.

**Figure A5:**
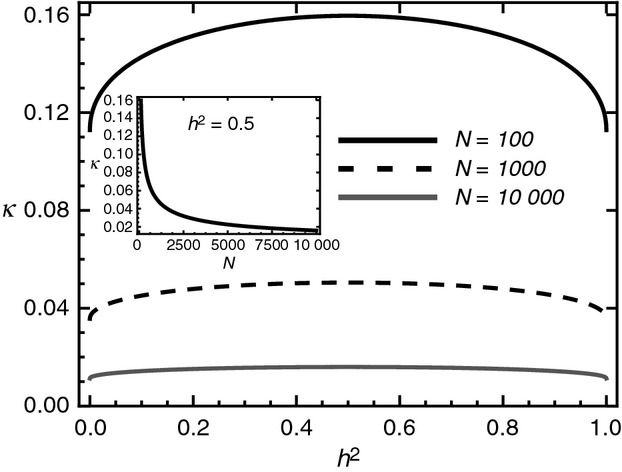
Expected absolute rate of phenotypic change *κ* between generations due to genetic drift and environmental variance, which contribute 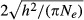 and 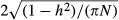, respectively (see main text), as a function of heritability *h*^2^ and population size *N* = *N*_*e*_ (inset). Environmental variance 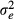 refers to the phenotypic variance caused by developmental instability and micro-environmental fluctuations.

## Appendix

### Appendix 1: Adaptation and extinction in the one-dimensional moving-optimum model

The following is a simplified version of the model by Bürger and Lynch (1995), which assumes the Gaussian fitness function

(A1)

with

(A2)

here, *z* is the phenotype of an individual, *w*_*z*,*t*_ its fitness at time *t*, *θ*_*t*_ is the optimal phenotype, which increases linearly at rate *k*, and *ω*^2^ measures the width of the fitness landscape (i.e., selection is strong if *ω*^2^ is small). *B* is the expected number of offspring (absolute fitness) of a perfectly adapted individual, and hence, ln *B* is the maximal population growth rate.

If the trait *z* is normally distributed in the population with mean  and variance , the mean absolute fitness at time *t* is

(A3)

with  describing the effective width of the fitness landscape (which is somewhat ‘smeared out’ by the environmental variance ). Equation (A3) shows that the maximal fitness *B* is reduced by two components of *genetic load*: The *standing load* (the square root term) due to standing genetic variation, and the *lag load* due to the deviation of the mean phenotype from the optimum.

For constant , a population with mean phenotype  evolves according to Lande's equation , where the directional selection gradient at time *t* is

(A4)

*β*_*t*_ measures the proportional change in log mean fitness per unit change of the mean phenotype.

As outlined in the main text, the population will reach a state of dynamic equilibrium, where it follows the optimum with a constant lag, which is given by

(A5)

(Bürger and Lynch 1995).

At the same time, the population dynamics are governed by  (e.g., Gomulkiewicz and Holt 1995) or a density-dependent version thereof (e.g., Bürger and Lynch 1995). In any case, population survival requires that, given the equilibrium lag , the equilibrium mean fitness . This condition yields the critical rate of environmental change

(A6)

(Bürger 2000), where the approximation is valid for weak selection (*V*_*s*_ ≥ 20; see Bürger 2000). When scaled by the phenotypic standard deviation, eqn (A6) gives the critical rate of phenotypic evolution

(A7)

in *haldanes*. Figure A1 illustrates the value of *κ*_crit_ as a function of heritability, the width of the fitness landscape and the reproductive potential of the population. The rule of thumb *κ*_crit_ ≤ 0.1 (Bürger and Lynch 1995) is based on *V*_*s*_ between 5 and 100, ln (*B*) < 1, and *h*^2^ < 0.5. Bürger and Lynch (1995) note that genetic drift and fluctuating selection might decrease *κ*_crit_ even further.

## Appendix 2: Adaptation and extinction in the multidimensional moving-optimum model

The multivariate version of model (A2) for *n* selected traits is

(A8)

where the fitness landscape is a bell-shaped ‘hill’, whose orientation and dimensions are determined by the (positive semi-definite) covariance matrix ***ω*** (of size *n*×*n*).

If the phenotype distribution is multivariate normal, the mean fitness is

(A9)

which, as in the univariate case, is reduced by a standing load (the square root term) and a lag load (the exponential).

The mean phenotype evolves according to the multivariate Lande equation , where the multivariate selection gradient is

(A10)

In the gradual-change scenario, ***θ***_*t*_ = **k***t*, the rate and the direction of environmental change is described by a speed vector **k**=(*k*_1_,…,*k*_*n*_)′, which contains the rates of change in the optimum for each trait. As in the univariate case, the population will eventually follow the optimum with a constant lag (assuming the **G**-matrix is constant):

(Jones et al. 2004; Gomulkiewicz and Houle 2009; Chevin 2013; Jones et al. 2012). Again, the population can persist if . If the vector **k** is decomposed into its length and its direction, **k** = ‖**k**‖**c** with **c** = **k**/‖**k**‖, then this condition is satisfied if

(A11)

(Gomulkiewicz and Houle 2009).

## Appendix 3: Fixation of a major mutation

Assume constant selection and a major mutation increasing fitness from 1−*s* to 1. Let the phenotypic effect of this mutation be *δz*. For simplicity, we look at the haploid case and neglect genetic variation at other loci as well as environmental variance. Then, the phenotypic variance of the population at time *t* is , where *p*_*t*_ is the frequency of the beneficial allele. The per generation change in mean phenotype is

(A12)

which in *haldanes* is

(A13)

independent of *δz*. The maximal rate of change, which is achieved when *p* = 1/2, is *s*/(2−*s*), which may be large if selection is strong (e.g., for *s* = 1/2, *κ*_max_ = 1/2).

## Appendix 4: Maximal sustainable rates of phenotypic evolution over ‘time frames of conservation interest’

Barrett and Hendry (2012) have argued that, over time frames of conservation interest, maximal sustainable rates of phenotypic evolution could well exceed the 0.1 *haldanes* that have been proposed by Bürger and Lynch (1995) for the long-term equilibrium of the moving-optimum model. Their point was that environments will not keep changing forever and that conservation biology is rather concerned with population survival over modest periods of time (e.g., 50 generations). Here, we attempt to evaluate this claim by calculating critical rates of environmental and phenotypic change, *k*_crit_(*t*_crit_, *N*_crit_) and *κ*_crit_(*t*_crit_, *N*_crit_), such that the population consists of *N*_crit_ individuals after *t*_crit_ generations. Note that, as we are no longer considering a dynamic equilibrium, the two rates *k*_crit_ and *κ*_crit_ are no longer equivalent. Our analysis is based on a ‘quasi-deterministic’ approximation developed by Bürger and Lynch (1995) for studying the mean time to extinction. This analysis neglects evolution of genetic variance in response to selection, as well as several sources of stochasticity (see Appendix 5). Its results can therefore only be a first approximation, which, however, help to elucidate several principals.

Consider, first, the case *N*_crit_ = *N*_0_, that is, we require that the population size does not decline from its initial value *N*_0_ over *t*_crit_ generations. The corresponding critical rate of environmental change can be calculated by rearranging equation (12a) in Bürger and Lynch (1995), which gives

(A14)

where *k*_crit_(∞, *N*_0_) is the critical rate given in eqn (A6) for infinite times. While *k*_crit_(*t*_crit_, *N*_0_) can substantially exceed *k*_crit_(∞, *N*_0_), the corresponding critical rates of phenotypic change are identical, that is, *κ*_crit_(*t*_crit_, *N*_0_) = *κ*_crit_(∞,*N*_0_) = *k*_crit_(∞, *N*_0_)/*σ*_*p*_. The reason is that both *κ*_crit_(*t*_crit_, *N*_0_) and *κ*_crit_(∞, *N*_0_) are achieved when the mean absolute fitness  (see eqn A3). This illustrates that eqn (A6) does not depend on the assumption of an indefinitely moving optimum and that the corresponding *κ*_crit_ simply gives the maximal rate at which the population can evolve without decreasing in size. In other words, rates of phenotypic evolution can only exceed *κ*_crit_(∞, *N*_0_) if population size declines.

To study this case, we now allow moderate population decline while still requiring the population size to remain above a critical threshold *N*_crit_ < *N*_0_ over *t*_crit_ generations. No analytical solution exists (Bürger and Lynch 1995), but the critical rates can be estimated numerically by iterating eqn (4). As a reduction in population size also entails a reduced genetic variance, we followed Bürger and Lynch (1995) by assuming that  at any time is given by the stochastic house-of-cards approximation for the current *N*.

Figure A2 shows maximal rates of environmental and phenotypic change under the constraint that the population is to maintain a minimal size of *N*_crit_ = 50 individuals over *t*_crit_ = 50 generations and compares the results to those from the Bürger and Lynch (1995) framework (no reduction in population size over infinite times) and those from eqn (A14) (no reduction in population size over 50 generations). The critical rate of environmental change, *k*_crit_, increases substantially when short-term reductions in population size are allowed (top row of Fig. A5). In contrast, the differences in the critical rates of phenotypic change, *κ*_crit_ are much less pronounced (bottom row), in particular for small populations (which might be of highest interest for conservation). Even in large populations, the relative increase in *κ*_crit_ rarely exceeds 30% (unless selection is extremely strong). Given the large uncertainty in our estimates of evolutionary rates (see below), this increase appears minor, and we conclude that considering adaptation over ‘time frames of conservation interest’ does not substantially alter the rule-of-thumb that critical rates are typically around 0.1 *haldanes*.

## Appendix 5: Stochastic fluctuations in evolutionary rates

The analysis presented in Appendix 4 was based on a deterministic approximation, which neglects various sources of stochasticity (see main text). To illustrate this stochasticity, we conducted individual-based simulations as described in Bürger and Lynch (1995). Two exemplary runs are shown in Fig. A3. While the population mean phenotype follows the moving optimum, generation-to-generation rates of phenotypic change (*κ*) in *haldanes* fluctuate as a consequence of nonselective factors such as genetic drift, environmental variance and fluctuations in the phenotypic variance . Observed fluctuations in *κ* are further amplified if only a part of the population is sampled (gray lines in Fig. A3), and their range can largely surpass the 0.1 *haldanes* predicted by Bürger and Lynch (1995), see Fig. A4. Similarly, in small populations, drift and environmental variance alone can induce rates of changes of up to 0.15 *haldanes* (Fig. A5). Overall, these results cast serious doubt on our ability to predict the fate of populations based on short-term measures of micro-evolutionary change.
